# The beneficial and detrimental effects of prolactin hormone on metabolic syndrome: A double‐edge sword

**DOI:** 10.1111/jcmm.70067

**Published:** 2024-12-11

**Authors:** Ayah Talal Zaidalkilani, Hayder M. Al‐Kuraishy, Ali I. Al‐Gareeb, Athanasios Alexiou, Marios Papadakis, Ammar AL‐Farga, Othman A. Alghamdi, Mostafa M. Bahaa, Mohammed Alrouji, Mohammed S. Alshammari, Gaber El‐Saber Batiha

**Affiliations:** ^1^ Department of Nutrition, Faculty of Pharmacy and Medical Sciences University of Petra Amman 11196 Jordan; ^2^ Department of Clinical pharmacology and Medicine College of Medicine, Mustansiriyah University Baghdad Iraq; ^3^ Department of Science and Engineering Novel Global Community Educational Foundation Hebersham New South Wales Australia; ^4^ AFNP Med Wien Austria; ^5^ University Centre for Research & Development Chandigarh University Mohali Punjab India; ^6^ Department of Research & Development Funogen Athens Greece; ^7^ Department of Surgery II University Hospital Witten‐Herdecke, Heusnerstrasse 40, University of Witten‐Herdecke Wuppertal Germany; ^8^ Department of Biochemistry College of Science University of Jeddah Jeddah Saudi Arabia; ^9^ Department of Biological Sciences College of Science, University of Jeddah Jeddah Saudi Arabia; ^10^ Pharmacy Practice Department, Faculty of Pharmacy Horus University New Damietta Egypt; ^11^ Department of Clinical Laboratory Sciences College of Applied Medical Sciences, Shaqra University Shaqra Saudi Arabia; ^12^ Department of Pharmacology and Therapeutics, Faculty of Veterinary Medicine Damanhour University Damanhour Egypt

**Keywords:** insulin resistance, metabolic syndrome, prolactin

## Abstract

The metabolic syndrome (MetS) is a clustering of abdominal obesity, hypertension, hyperglycaemia, hypertriglyceridemia and low high‐density lipoprotein (HDL) level. MetS development is affected by endocrine hormones such as prolactin (PRL) hormone which induce insulin resistance and central obesity because PRL is implicated in the pathogenesis of MetS. Pituitary PRL controls mammary gland, however extra‐pituitary PRL is highly intricate in the regulation of adipose tissue function. In addition, cAMP activators enhance expression and release of PRL which involved in the control of lipogenesis and energy homeostasis. Consequently, hyperprolactinaemia may be associated with the development of MetS. However, normal physiological level of PRL is essential for insulin sensitivity and regulation of adipose tissue function and energy metabolism. Therefore, PRL has dual effects on the components of MetS. Hence, the present review aims to discuss the modulatory mechanistic role of PRL on MetS regarding its beneficial and detrimental effects.

## 1 INTRODUCTION

The metabolic syndrome (MetS) is a well‐defined as a clustering of different components including abdominal obesity, hypertension, hyperglycaemia, hypertriglyceridemia and reduce high‐density lipoprotein (HDL) level.^1^, ^2^ The worldwide prevalence of MetS is about 30% that increases mainly in the US population and may reach to 34%.^3^ MetS increases risk for the development of Type 2 diabetes (T2D) and cardiovascular disorders.^1^ MetS, insulin resistance (IR) and prediabetes are closely interrelated and have overlapping pattern. It has been reported that MetS is caused by disorders of energy storage and utilisation that is an interesting area of ongoing research.^4^

It has been reported that MetS was first recognized by Joslin in 1921 who find a link between T2D, hyperuricemia, and hypertension. Kylin in 1923 described the mechanistic association between T2D, hyperuricemia and hypertension.^5^ In 1967, the term MetS was first used by Avogadro and co‐workers who described obese patients with dyslipidemia.^6^ In 1978, Gerald reported that MetS as a potential risk factor for the development of myocardial infarction and syndrome X due to the development of IR.^7^

The pathophysiology of MetS is complex and related to defect in the differentiation of adipocytes and formation of visceral fat which induce the release of pro‐inflammatory cytokines such as tumour necrosis factor alpha (TNF‐α) which induces the development of IR.^8^

**FIGURE 1:**
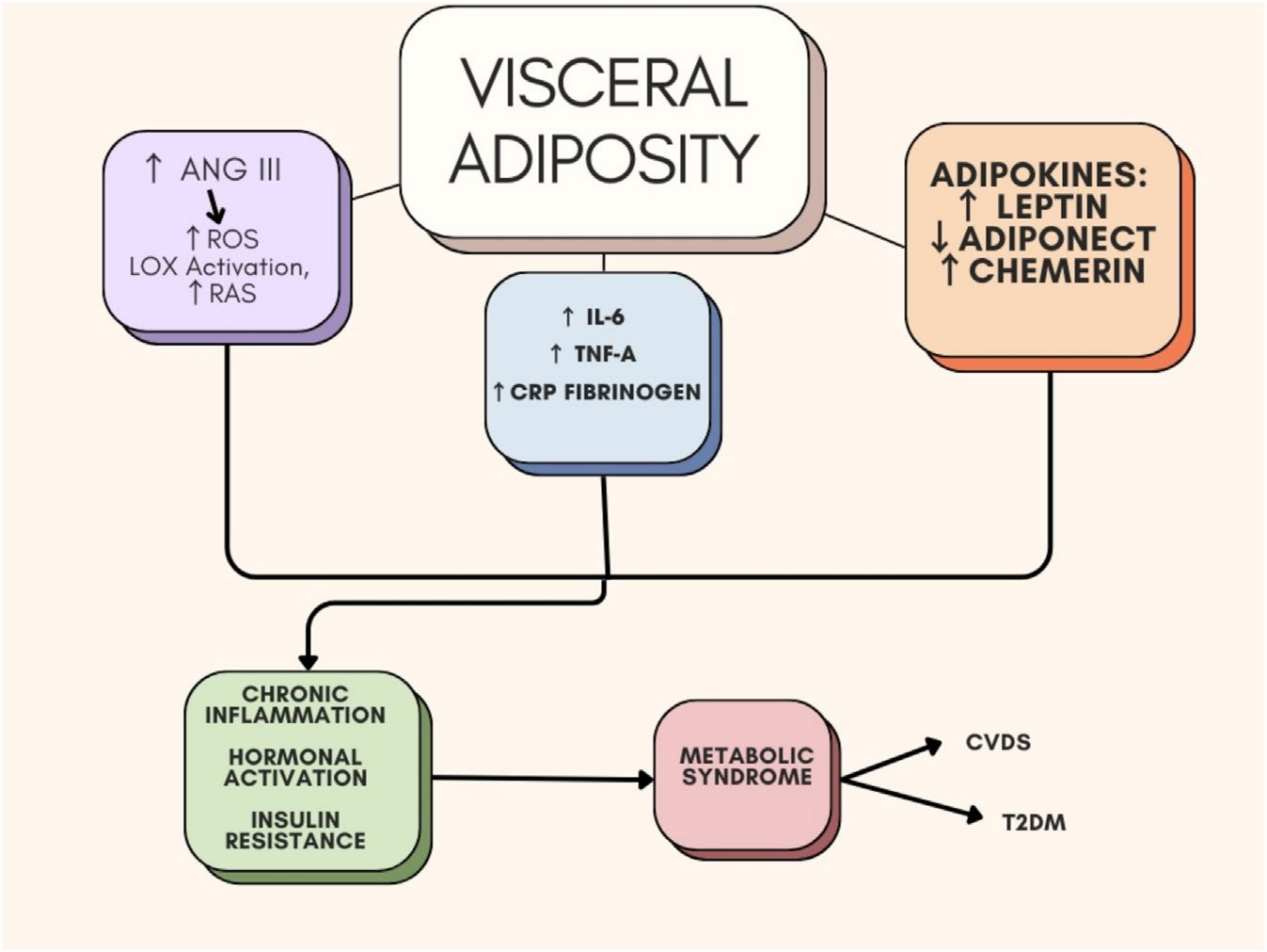
Pathogenesis of MetS. Visceral adiposity increases the activation of renin‐angiotensin system (RAS) mainly angiotensin II (AngII) which induce the formation of reactive oxygen species (ROS) and lipooxygenase (LOX). As well, visceral adiposity increased the release of pro‐inflammatory leptin and chemerin, and reduced the release of anti‐inflammatory adiponectin. Furthermore, visceral adiposity promotes the release of pro‐inflammatory cytokines such as IL‐6, tumour necrosis factor alpha 1 (TNF‐α1), CRP and other factors such as fibrinogen. These changes induced by visceral adiposity cause chronic inflammation, hormonal changes and insulin resistance (IR).

MetS model is induced by fed with 33% sucrose which increase triglyceride level and induce visceral fat accumulation and development of IR.^8^ In addition, visceral fat‐induced inflammation promotes chronic inflammation which implicated in the pathogenesis of atherosclerosis, hypertension, and T2D.^9^ Multiple acquired and genetic factors are involved in the development of low‐grade inflammation and IR contribute in the development and progression of MetS.^10^ Increasing visceral fat mass exaggerates the activity of renin‐angiotensin system (RAS) causing inflammation and oxidative stress via induction the generation of reactive oxygen species.^11^ As well, visceral adiposity promotes the release of pro‐inflammatory cytokines and alteration expression of adipocytokines by increasing inflammatory leptin and chemerin, and reduction of anti‐inflammatory adiponectin. Furthermore, visceral adiposity increases circulating fatty acids leading to reduction of peripheral glucose utilisation and enhances gluconeogenesis causing hyperglycaemia and IR.^12^ Collectively, expansion of visceral fatty mass is the primary events in the pathogenesis of MetS (Figure 1).

Moreover, brain is also involved in the pathogenesis of MetS by modulating peripheral lipid and carbohydrate through endocannabinoid system which promotes CNS activation and food intake leading to MetS.^13^ Endocannabinoids through activation of cannabinoid type 1 receptor (CB1R) promotes CNS activation and food intake leading to MetS; therefor inhibition of CB1R by selective blocker is used in treating obesity and MetS.^13^ Remarkably, inhibition of peripheral CB1R only by molecules improves IR, obesity, fatty liver and other components of MetS.^13^ Furthermore, overfeeding with fructose or sucrose in attendant with high‐fat diet leads to the development of MetS In addition, high consumption of fatty acids promotes generation of arachidonic acid (AA) which involved in the pathogenesis of MetS by inducing inflammatory reactions.^14^ AA derivative diacylglycerol enhances formation of 2‐arachidonoylglycerol (2‐AG) which promote eicosanoid synthesis.^14^

The causes of MetS are different including chronic stress, consumption of sugar sweetener beverage, aging, sedentary life, genetic factors, sleep disorders and excessive alcohol consumption. However, there is a potential debate whether IR and obesity are consequences or causes of MetS. Of note, T2D is regarded as a complication of MetS due to reduction of adiponectin.^15^ Notably, western diet habit and linked weight gain promotes the development of MetS. Abdominal obesity enhances macrophage activity and polarisation toward inflammatory one with subsequent release of pro‐inflammatory cytokines and chemokines.^16^ Therefore, anti‐inflammatory and insulin sensitising agents have ability to abrogate the link between inflammation and IR in MetS.^16^ It has been shown that chronic stress induces dysfunction of hypothalamic–pituitary adrenal (HPA) axis leading to elevation of cortisol which causes IR and abdominal adiposity.^17^ Besides, aging enhances the development of MetS mainly in women.^18^ Furthermore, lipodystrophic disorders and rheumatic diseases increase risk of MetS through induction of IR.^19^ Remarkably, MetS is associated with increased risk of chronic obstructive pulmonary disease (COPD).^20^ It has been shown that 50% of patients with COPD have MetS as comorbidity. Though, it is presently not clear whether MetS is the cause or as a consequence of COPD.^20^

## 2 THE DIAGNOSIS OF MetS IS ACCORDING TO THE SPECIFIC DIAGNOSTIC CRITERIA

Furthermore, MetS development is highly affected by endocrine hormones such as prolactin (PRL) hormone which affect IR and central obesity. Different studies implicate PRL in the pathogenesis of MetS (Table 1).^26^, ^27^ Of note, pituitary PRL mainly regulates mammary gland, though extra‐pituitary PRL is highly involved in the regulation of adipose tissue function. As well, cAMP activators enhance the expression and release of PRL which involved in the control of lipogenesis and energy homeostasis.^27^ Therefore, hyperprolactinaemia may be linked with the development of MetS. However, normal physiological level of PRL is essential for insulin sensitivity and regulation of adipose tissue function and energy metabolism.^28^ Thus, the present review aims to clarify the modulatory role of PRL on MetS regarding its beneficial and detrimental effects.

**TABLE 1:** Diagnostic criteria of MetS.

Federation and association	Diagnostic criteria	Reference
IDF (international diabetes federation)	TG level >150 mg/dL HDL <40 mg/dL SBP >130 mmHg, DBP >85 mmHg FBG >100 mg/dL	^21^
WHO (World Health Organization)	TG level >150 mg/dL BP >140/90 mmHg Central obesity Microalbumuria >20 μg/min	^22^
EGIR (European Group for insulin resistance)	BP >140/90 mmHg Central obesity TG level >150 mg/dL FBG >100 mg/dL	^23^
NCEP (National Cholesterol Education Program)	Central obesity TG level >150 mg/dL HDL <50 mg/dL BP >140/90 mmHg FBG >110 mg/dL	^24^
AHA (American Heart Association)	Central obesity TG level >150 mg/dL HDL <40 mg/dL BP >130/85 mmHg FBG >100 mg/dL or use medication for hyperglycaemia	^25^

## 3 OVERVIEW OF PRL PHYSIOLOGY

PRL is also known as lactogenic hormone (lactotropin) is a peptide hormone released from anterior pituitary gland intricate in milk production in women and control of sex drive and sexual functions in both sexes.^29^ PRL release from anterior pituitary is inhibited by dopamine and stimulated by thyrotropin releasing hormone (TRH) from hypothalamus. In addition, PRL‐releasing peptide (PrRP) was first isolated from bovine hypothalamus as an orphan G‐protein‐coupled receptor. The early studies disclosed that PrRP was a potent and specific PRL‐releasing factor. Morphological and physiological studies, however, indicated that PrRP may play a wide range of roles in neuroendocrinology other than PRL release, including metabolic homeostasis, stress responses, cardiovascular regulation, gonadotropin secretion, growth hormone (GH) secretion and sleep regulation.^30^

Furthermore, PRL secretion from anterior pituitary is stimulated by other hormones and neurotransmitters.^31^ PRL release is also activated by vasoactive intestinal polypeptide, oestrogen, and breast suckling.^32^ It has been shown that circulating PRL is present in three forms; big‐big PRL (150 kDa), big‐PRL (48 kDa) and little‐PRL (22 kDa). Both big‐big PRL and big‐PRL have little biological activity while little‐PRL is the most active circulating one.^33^

PRL acts on PRL receptors (PRLRs) which also receptor for colony‐stimulating factor, erythropoietin and interleukin 6 (IL‐6). PRLRs are considered as functional receptor for placental lactogen and GH.^34^ Prominently, PRL may intricate in the regulation of immune function by modulating expression of cytokines different isoforms of PRLRs according to their size are present including large PRLR (PRLR‐L), intermediate PRLR (PRLR‐I) and small PRLR (PRLR‐S).^34^ PRLRs are highly expressed in various tissues including endocrine organs and immune cells such as microglia and monocytes. Furthermore, PRL hormone has diverse pleiotropic effects such improvement of neuronal myelination, neurogenesis and fetal lung maturation during pregnancy.^35^ Indeed, extra‐pituitary PRL which released from immune cells, myometrium, and breast has autocrine and paracrine function that not affected by dopamine inhibitory effects.^36^

Regarding PRL serum level, PRL during the fetal life start to increase during 20–24 weeks, and continue to elevated from 30 week and reach the maternal level.^37^ During the postpartum period, PRL serum level is increased by 10 fold and then reaches to the normal level at 3rd month of life.^38^ During aging PRL serum level is dramatically reduced to reach half of that present in young age.^39^ In women, PRL serum level is increased at mid‐cycle and reduced at follicular phase due the effect of gonadotropin‐releasing hormone. Nevertheless, PRL serum level remains elevated till 46 weeks postpartum.^40^ Noteworthy, PRL serum level is higher during sleep and morning, augmented by emotional stress, exercise, protein diet and after epileptic seizure. It has been shown that PRL serum level in epileptic seizure but normal in psychogenic seizure, and in this way can differentiate between the mentioned seizures.^41^ Numerous drugs may increase PRL levels such as antipsychotics which are often responsible for drug‐related hyperprolactinaemia.^42^ It has been stated that PRL serum level is increased in various pathological conditions as in pituitary prolactinoma, adrenal failure, and primary hypothyroidism.^43^ Risperidone is one of the atypical neuroleptics can induce hyperprolactinaemia, while other atypical drugs are infrequently or transiently increase of PRL levels. Women are more sensitive than men to the hyperprolactinaemic effect of antipsychotics.^42^ Risperidone‐induced hyperprolactinaemia may be reverted discontinuation of this drug. Antidepressant drugs including selective serotonin reuptake inhibitors, monoamine oxidase inhibitors and some tricyclics can cause hyperprolactinaemia.^42^

It has been stated that PRL serum level is increased in various pathological conditions as in pituitary prolactinoma, adrenal failure, and primary hypothyroidism.^43^ Hyperprolactinaemia is regarded when PRL serum level is increased more than 20 ng/mL in men and 25 ng/mL in women.^44^ Moreover, higher PRL level inhibits the release of oestrogen in women and testosterone in men, though physiological level of PRL activates testosterone secretion from Leydig cells and improve spermatogenesis. PRL has a short half‐life; it is metabolized by liver and excreted by kidney, thus PRL serum level is augmented in chronic renal failure.^42^ PRL triggers activation of CRH neurons by inducing the expression of JAK/STAT pathway.^45^

These observations indicated that PRL is not only lactogenic hormone, but has pleiotropic effects on different organ system. Therefore, hyperprolactinaemia may affect the metabolic profile and immune system in bidirectional ways.

## 4 METABOLIC ROLE OF PRL

**FIGURE 2:**
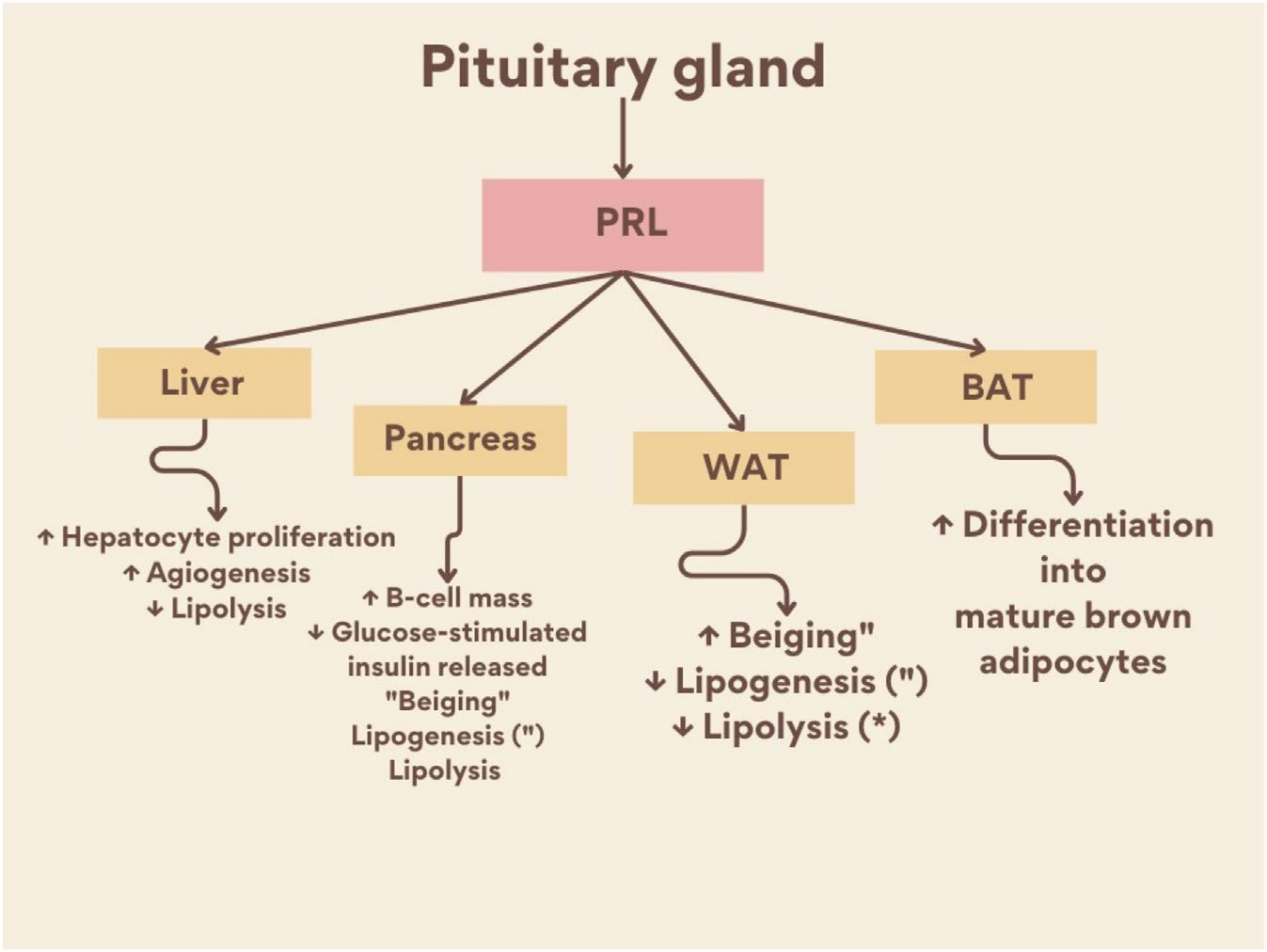
Metabolic effect of PRL. Prolactin (PRL) increases hepatocyte proliferation and angiogenesis and inhibits lipolysis in liver. PRL acts on the pancreas by increase pancreatic β‐cell mass, and modulates glucose‐stimulated insulin release. On white adipose tissue (WAT) PRL regulates adipogenesis and lipolysis. On brown adipose tissue (BAT) increases the differentiation into mature brown adipocytes.

It has been observed that PRL at physiological level has numerous metabolic effects by controlling of energy balance and cellular metabolism in the brain and peripheral organs.^46^ PRL improves liver function by increasing angiogenesis and inhibiting lipolysis.^46^ STAT5 activation is necessary for migration and invasion of endothelial cells. STAT5 effects on endothelial cells require the secretion of PRL which induce activation of MAPK and STAT5. In vivo, endothelial cell‐derived PRL is expected to be involved in the tumour microenvironment. Thus, PRL may stimulate tumour angiogenesis via autocrine, paracrine, and endocrine pathways. The interruption of tumour angiogenesis by inhibiting of PRL signalling may be a striking target for therapeutic interpolation.^47^ In addition, PRL regulates the metabolic function of pancreas and adipose tissues (Figure 2).

The metabolic effect of PRL was first reported in 1949 by Bernado A. Hussey who describes the diabetogenic effect of PRL in animal model study.^48^ The metabolic effect of PRL is not limited to glucose homeostasis, but control endothelial function and lipid metabolism. It has been shown that pathological PRL level is linked with cardiovascular complications due to alteration of lipid metabolism and development of endothelial dysfunction in patients with chronic kidney disease.^49^ PRLRs in pancreatic β cells improve insulin release in response to blood glucose at physiological PRL level, though hyperprolactinaemia can induce hyperglycaemia and IR.^49^ Controlling and treatment of hyperprolactinaemia lead to considerable mitigation of hyperglycaemia and IR.^37^ Dopamine agonists are usually used in neurological disorders like Parkinson's disease, restless leg syndrome, and hyperprolactinaemia. However, dopamine agonists also effective in reducing the glycaemic level in T2D patients by improving glycaemic levels, and reducing free fatty acids (FFA) and triglycerides. Cabergoline reduces glycated haemoglobin by 0.4%–0.8% and cardiovascular risk in IR patients.^37^ Dopamine agonists improve hypothalamic dopamine tone and prevent extreme sympathetic tone within the central nervous system (CNS), resulting in a reduction in postprandial blood glucose levels by enhancing suppression of hepatic glucose production. Bromocriptine also reduces fasting and postprandial plasma free fatty acid and triglyceride levels. In a double‐blind, placebo‐controlled study in T2D patients, bromocriptine reduced cardiovascular end points, though the mechanism of these agents on cardiovascular disease was not fully elucidated.^47^

Conversely, low PRL level increases T2D risk by reducing insulin release and insulin sensitivity.^50^ A cohort study involved adipose tissue samples from 40 T2D patients showed that low PRL serum level was associated with visceral adipocyte hypertrophy and IR signifying that low physiological PRL level may increase T2D risk. Therefore, PRL serum level has a U shape effect on blood glucose and insulin sensitivity. However, this effect was not observed in relation to lipid profile, endothelial function, and bodyweight.^50^ In this state; Homeo‐FIT‐PRL concept is suggested by recent studies to explain the metabolic adaptation in response to high metabolic demands.^51^ Thus, PRL may has a dual effect could be beneficial or detrimental.

## 5 BENEFICIAL EFFECT OF PRL ON MetS

### 5.1 Preclinical findings

Various preclinical studies highlighted that moderately elevated PRL level has a beneficial role against metabolic disorders. Progressive accumulation of visceral fat provokes IR and other features of MetS.^52^ Of note, MetS, obesity and T2D are associated with significant reduction of circulating PRL level. It has been shown that administration of PRL via mini‐pumps attenuate IR and reduces inflammatory cytokines and retard adipocyte hypertrophy in visceral fat of diet‐induced obesity in rats.^52^ In PRLR knockout mice with high‐fat diet induces severe IR, glucose intolerance and visceral fat hypertrophy compared to wild‐type mice.^52^ Similarly, deletion of PRLRs exacerbates streptozotocin (STZ)‐induced IR and hyperglycaemia in diabetic mice.^53^ Besides, selective deletion of PRLRs in pancreatic β cell triggers the development of gestational diabetes in pregnant mice.^54^ In addition, administration of analogue PrRP analogue reduces obesity and IR in mice with induced obesity.^55^ Of note, PrRP which act on G‐protein coupled receptor 10 (GPR10) is released from hypothalamus to activate the release of PRL from anterior pituitary.^56^ PrRP and GPR10 deficient mice are at high propensity to develop obesity and IR due to lowering of circulating PRL level.^56^ Therefore, lipidized PrRP could be a potential treatment of IR, obesity and T2D.

On the other hand, Park et al., observed that administration of intraperitoneal PRL at low dose (25 μg/kg) or high dose (250 μg/kg) each 12 h, promote the proliferation of pancreatic β cells by activating signal transducer and activator of transcription 5 (STAT5) in diabetic rats.^2^ PRL promotes glucose homeostasis by increasing β‐cell mass under certain conditions such as pregnancy, whereas hyperprolactinaemia due to a pituitary gland adenoma tumour exacerbates IR. High PRL level aggravate IR and impair the insulin‐secretory capacity in diabetic mice.^2^ Pancreatic β cells adapt to IR during pregnancy by up regulating β‐cell mass and increasing insulin secretion. PRL signalling affects β‐cell development via activations of PRLRs which found inside and outside of pancreatic β‐cells.^57^ A recent experimental study illustrated that down regulation of PRL action during pregnancy in non‐β‐cells negatively affects β‐cell gene expression, and increased β‐cell susceptibility to external insults such as glucolipotoxicity.^57^ Therefore, development of PRLR agonists may have beneficial effects in the management of human T2D.

Remarkably, maternal hypoprolactinaemia and early weaning increase risk for the development of MetS in offspring.^58^ Supporting to this notion, an experimental study demonstrated that inhibition of PRL in lactating Wistar rats by bromocriptine, their offspring developed MetS after 180 days of life.^58^ A systematic review and meta‐analysis involved preclinical studies confirmed that hypoprolactinaemia increases risk of MetS and metabolic derangement.^59^ Therefore, normal physiological PRL level seems beneficial against IR, obesity and development of MetS.

### 5.2 Clinical findings

It has been shown that PRL leads to beneficial effects against different components of MetS in humans. PRL improves metabolic homeostasis, and defect in PRL/PRLRs axis induce metabolic disorders and systematic complications.^60^ For example, low PRL triggers the development of MetS in humans, and obese patients with high PRL level tend to have superior metabolic profile compared with those of low PRL level.^60^ A cross‐sectional study included obese patients following laparoscopic sleeve gastrectomy showed that lipid profile and glucose indices were better in patients with high PRL level as compared to patients with low PRL level.^61^ As well, PRL level was negatively correlated with waist‐hip ratio, neck circumference and blood pressure, and positively correlated with basal metabolic rate in men only.^61^ Furthermore, adequate high PRL level reduces T2D risk for about 10 years.^62^ A longitudinal study conducted by Li et al., illustrated those T2D free women followed for 22 years with baseline high PRL were inversely associated with incidence of T2D.^62^ Likewise, high PRL level is negatively associated with blood glucose and HbA1c in both women and men. A cross‐sectional study from China included 2377 subjects (1034 men and 1343 women) revealed that high PRL level reduced incidence and prevalence of impaired glucose tolerance and T2D.^63^ A systematic review and meta‐analysis revealed that low PRL level in T2D patients is associated with development of non‐alcoholic fatty liver diseases suggesting that PRL has a protective effect against the pathogenesis of non‐alcoholic fatty liver diseases.^64^

Evidence from patients‐based studies proposed that increasing dopamine in schizophrenic patients reduced PRL and increase T2D. Therefore, antipsychotic agents which inhibit dopamine, promote PRL serum level and reduce T2D risk.^65^

Interestingly, high PRL level during pregnancy attenuates the development of postpartum T2D though reduced PRL level in pregnant women with gestational diabetes is regarded as a predictive risk factor for the development of T2D within 10 years.^66^ PRL level during pregnancy is higher in women with normal glucose tolerance compared with women develop postpartum T2D. PRL is essential for regulation of pancreatic β cell function during pregnancy; therefore, reducing of PRL in women with gestational diabetes is linked with future pancreatic β cell dysfunction and development of T2D since PRL enhance insulin signalling and sensitivity.^66^

Another feature of MetS is IR which developed due to adipose tissue dysfunction and pancreatic β cell dysfunction, is linked with PRL level and signalling. Therefore, PRL is considered as a biomarker of adipose tissue dysfunction and IR, and medications that increase PRL level could be a novel therapeutic strategy in the management of T2D. It has been shown that PRL improves function of adipose tissue and attenuates accumulation of visceral adipose tissue by increasing adiponectin in women with polycystic ovary.^67^

Of note, hypertension is regarded as a cornerstone in the diagnosis of MetS. Physiological PRL level improves endothelial function through modulation of nitric oxide synthase (NOS).^68^ However, high PRL level is linked with post‐menopausal hypertension.^69^ Thus, the potential role of PRL concerning hypertension is controversial.

In the context of MetS, different studies revealed that high PRL level reduced the prevalence of MetS.^70^ A case control study on 94 obese children and 40 matched healthy children showed that PRL level was lower in obese children compared to healthy children.^70^ Low PRL level in obese children predicts the development of MetS.^70^ As well, low PRL level in men with erectile dysfunction is linked with high risk of MetS. PRL level is inversely correlated with dyslipidemia which is a critical component of MetS.^71^ In addition, high PRL level is linked with significant reduction of pro‐inflammatory cytokines such as TNF‐α which is involved in the pathogenesis of MetS.

**FIGURE 3:**
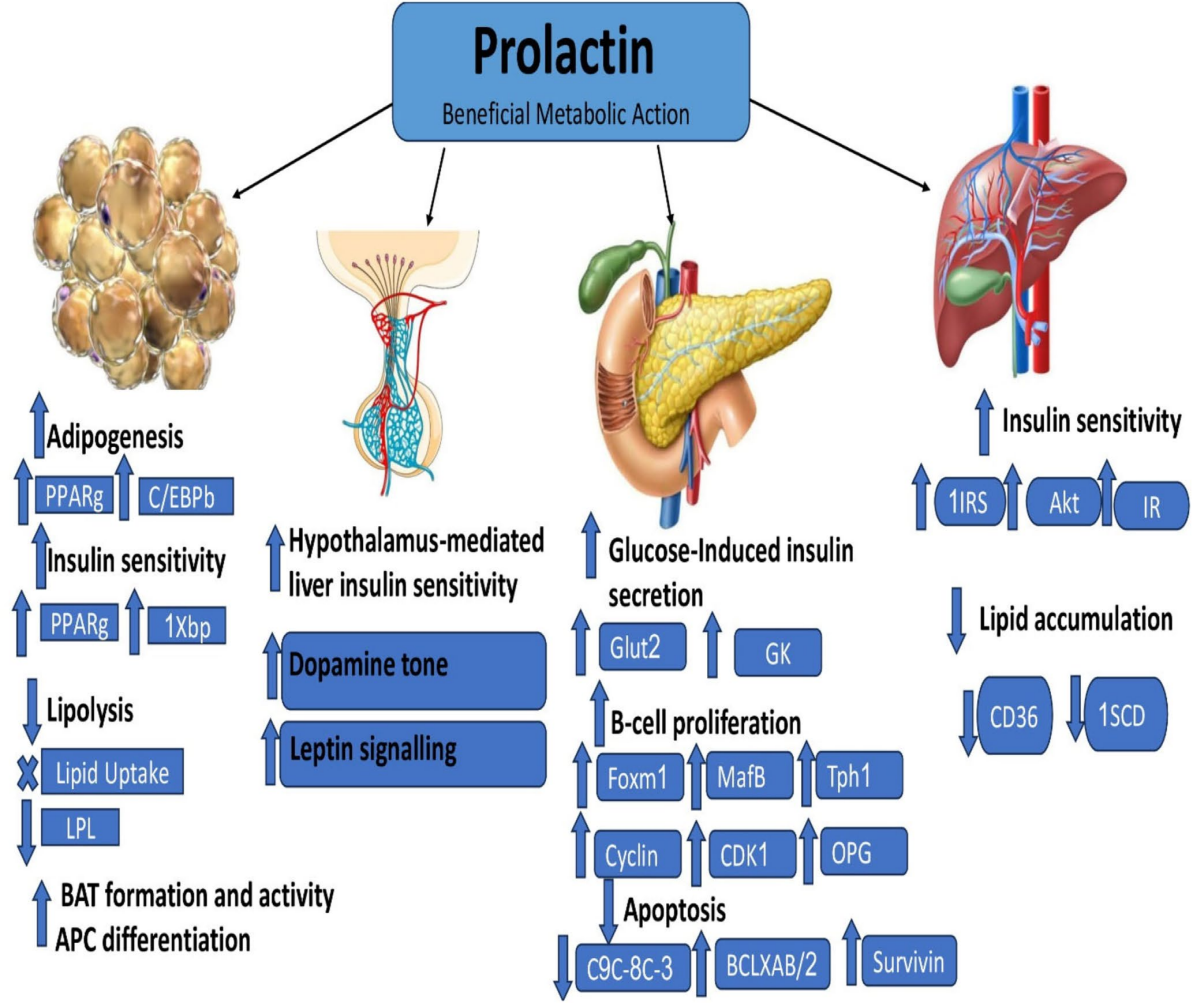
The beneficial effects of PRL against MetS.

In sum, PRL has a protective effect against the development and progression of MetS by various mechanisms (Figure 3).

## 6 DETRIMENTAL AND NEUTRAL EFFECTS OF PRL ON MetS

### 6.1 Preclinical findings

It has been reported that PRL deficiency had little effect on body weight, body composition, and lipid profile and adiponectin level in mice. In addition, PRL has negligible role on adipose tissue explants and lipolysis signifying that PRL has minor metabolic effect. In vitro and in vivo studies demonstrated that PRL inhibits release of adiponectin in both human adipose tissue and in mice. Reduction of adiponectin promotes the development of IR.^72^ In PRL‐overexpressing female mice, a minor decreasing effect in adiposity was recognized with no or little difference in body weight. In PRL‐deficient mouse model there was substantial reduction in bodyweight and visceral fat mass.^73^ Inhibition of PRL by bromocriptine in mice caused no changes in body weight and blood glucose. Though, increasing PRL in rats promote food intake and weight gain which reversed by bromocriptine.^74^ Furthermore, PRL is linked with the development of MetS by inducing IR and visceral obesity in animal model studies.^26^, ^27^ Moreover, hyperprolactinaemia has been illustrated to induce IR in animal model study in T2D rats.^75^ Increasing of circulating PRL can trigger the development of MetS in diabetic rats by reducing hypothalamic dopaminergic neurotransmission and subsequent abnormal lipogenic responsiveness to the effect of peripheral insulin. Furthermore, activation of hypothalamic dopaminergic neurotransmission by dopamine agonists can mitigate MetS in rats by reducing hepatic inflammation and IR by modulating liver gluconeogenic activity and lipogenesis.^76^ However, dopamine agonist quinpirole, and L‐dopa prevent glucose‐stimulated insulin secretion in human pancreatic β cells in preclinical studies.^77^ Furthermore, preclinical finding indicated that hyperprolactinaemia triggers weight gain and obesity in mice by affecting of hypothalamic orexigenic/anorexigenic balance.^78^ These preclinical findings proposed PRL excess can provoke metabolic disturbances and may lead to the development of MetS.

### 6.2 Clinical findings

**FIGURE 4:**
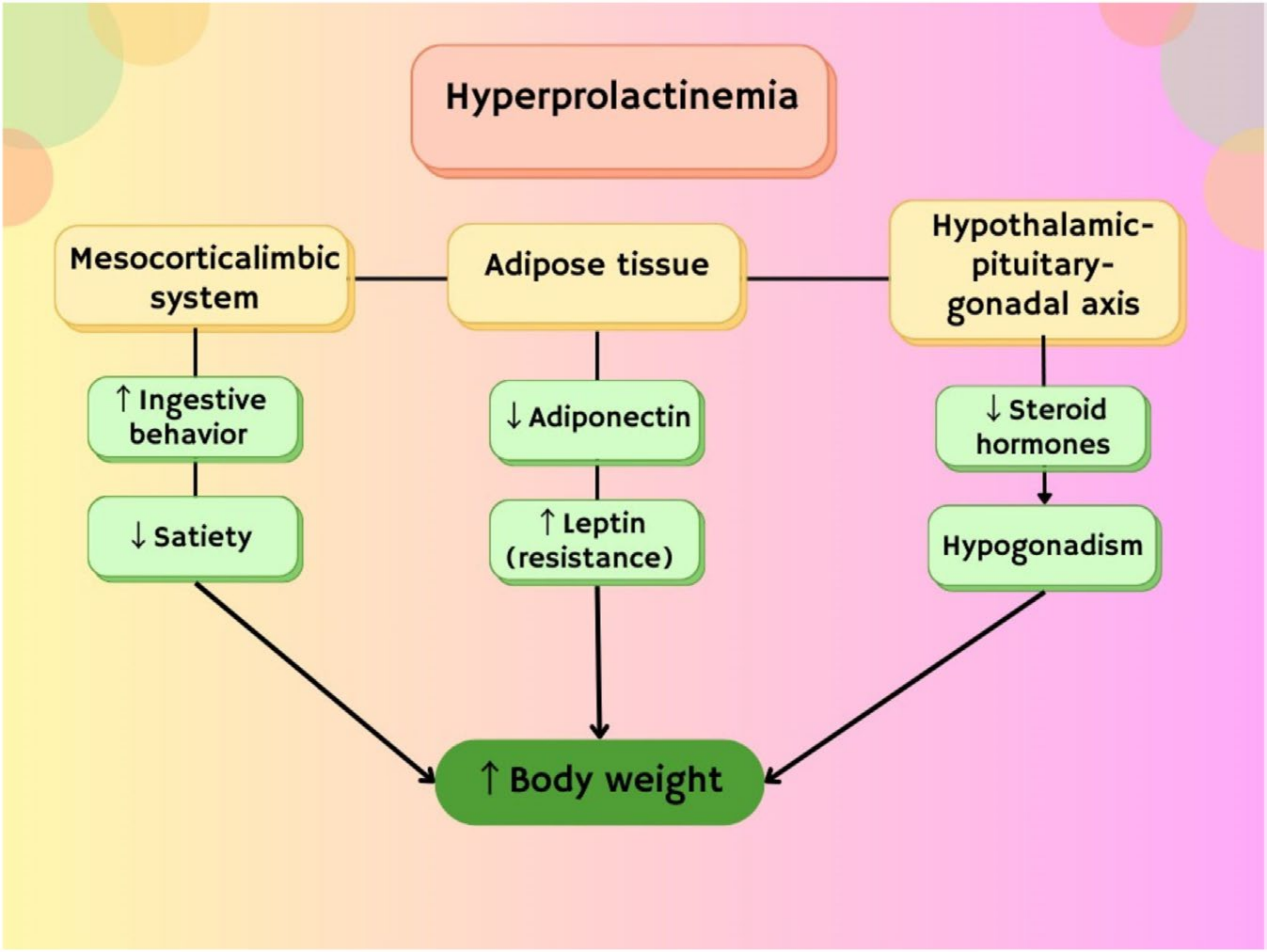
Hyperprolactinaemia and increasing of body weight.

Alongside, many clinical studies revealed that PRL excess may associated with the development of MetS by increasing of body weight and induction of IR. However, a cross‐sectional study discovered no clinical significance association between PRL serum level and risk of T2D and MetS.^79^ It has been revealed that PRL was not involved in the pathophysiology of obesity as confirmed by a cross‐sectional study which showed no link between obesity degree and metabolic derangement.^80^ Indeed, chronic PRL excess has been linked with increasing of food intake and weight gain leading to obesity and IR.^78^ Increasing appetite in patients with hyperprolactinaemia is attributed to the reduction of hypothalamic dopaminergic neurotransmission which involved to appetite inhibition and regulation of energy expenditure.^78^ As well, hyperprolactinaemia can induce the expression of neuropeptide Y (NPY), agouti‐related peptide (AGRP) and corticotrophin releasing hormone (CRH) which increase appetite and body weight.^78^ Therefore, body fat percentage is increased in patients with hyperprolactinaemia as compared to healthy controls.^81^ In addition, hyperprolactinaemia promotes expansion of visceral fat and epicardial adipose tissue thickness in patients with prolactinoma. Thus, patients with prolactinoma tend to have high waist circumference and BMI compared to healthy controls. These findings proposed that PRL excess is independent risk factor for obesity. Thus, restoration of normal PRL levels by dopaminergic agonists has been suggested by different studies to be substantial in the management of obesity.^82^ However, dopaminergic agonists may produce neutral effects on body weight in patients with hyperprolactinaemia signifying that obesity is mainly related to PRL excess rather than dopaminergic tone. Supporting to this notion, hyperprolactinaemia and associated hypogonadism further increase body weight as compared to non‐hypogonadal patients with hyperprolactinaemia as well as increased risk for MetS.^83^ Testosterone replacement therapy reduced body weight by 5%. Hence, improvement of PRL level can ameliorate bodyweight and MetS through regulation of testosterone level. Therefore, hyperprolactinaemia is connecting with increasing of body weight, one of the main components of MetS. The underlying mechanisms for increasing body weight are related to alterations mesocortical‐limbic system, development of leptin resistance and reduction of adiponectin release from adipose tissue.^84^ As well, hyperprolactinaemia inhibits hypothalamic–pituitary‐gonadal axis leading to the reduction of gondotrophin releasing hormone (GnRH), pituitary luteinizing hormone (LH) and follicular stimulating hormone (FSH) with subsequent decreasing of sex steroid hormones and development of hypogonadism^85^ (Figure 4).

**FIGURE 5:**
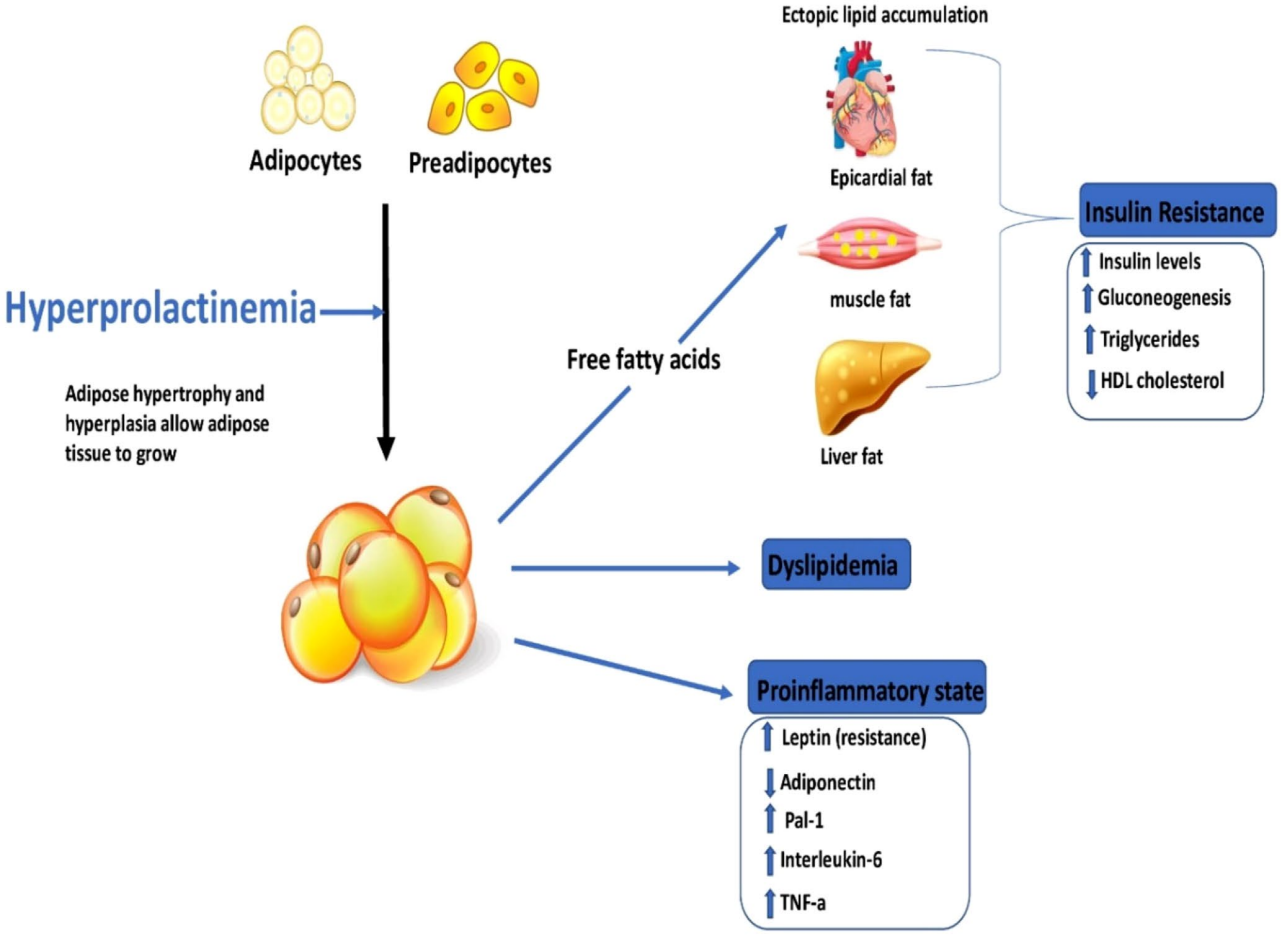
Chronic hyperprolactinaemia and cardiovascular complications.

Moreover, many studies indicated that high PRL serum level is connected with major cardiovascular complications such as myocardial infarction and atherosclerosis. Chronic hyperprolactinaemia activates adipose tissue leading to an increasing in circulating free fatty acids (FFAs) which cause IR, dyslipidemia and the release of pro‐inflammatory cytokines with subsequent development of cardiovascular complications^86^ (Figure 5).

**FIGURE 6:**
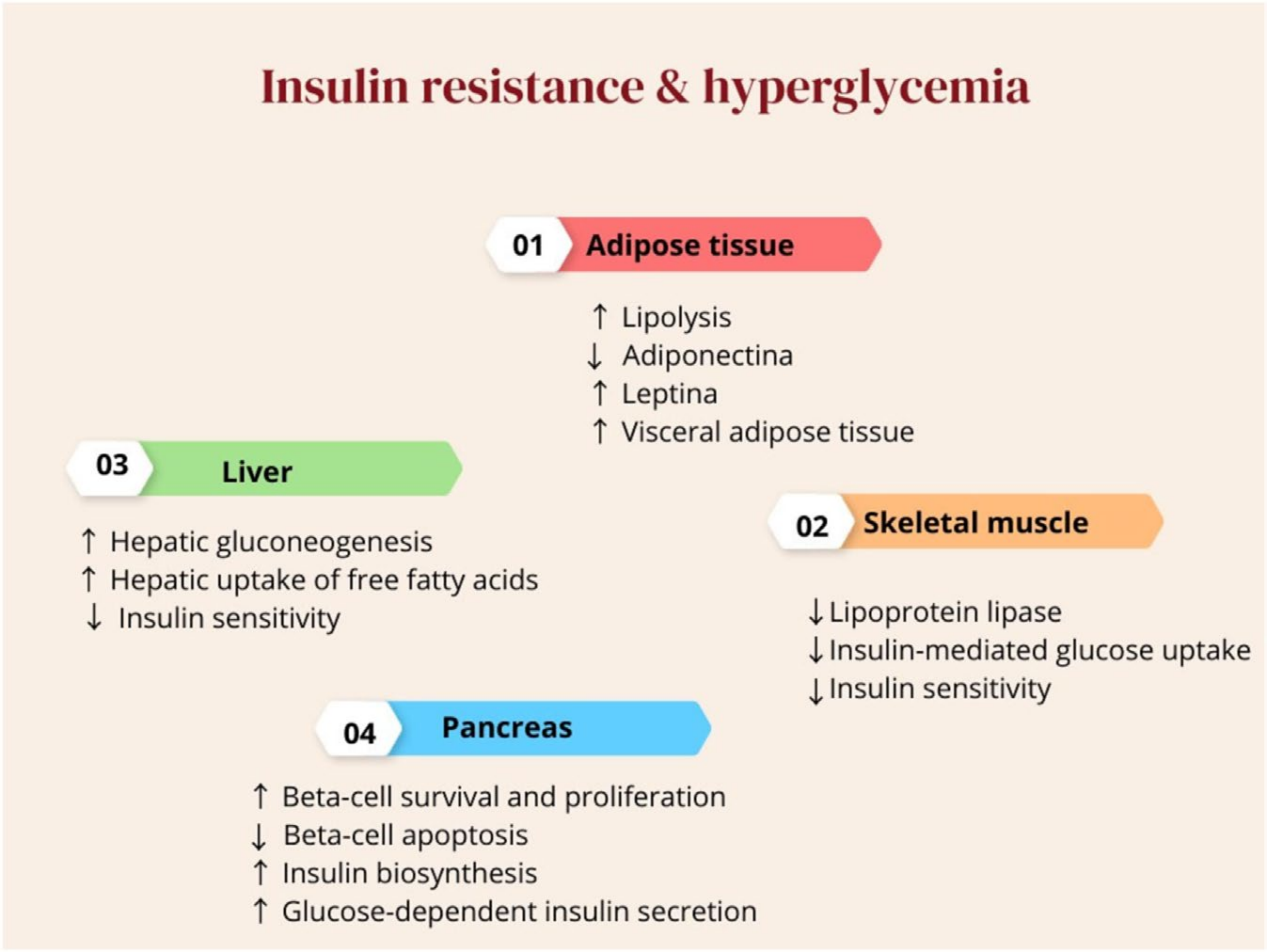
Chronic hyperprolactinaemia, hyperglycaemia, and IR.

Furthermore, chronic hyperprolactinaemia is associated with impaired insulin secretion and IR in many clinical studies.^87^, ^88^ Therefore, high circulating PRL in patients with prolactinoma can induce metabolic disturbance and development of MetS. Notably, PRL level within or above normal range may cause MetS.^87^ PRL excess enhances the development of MetS in one third of patients with hyperprolactinaemia due to suppression of central dopaminergic tone. Thus, activation of hypothalamic dopaminergic neurotransmission by dopamine agonists can reduce incidence and prevalence of MetS in patients with hyperprolactinaemia by controlling of PRL release and improving of insulin sensitivity.^88^ In addition, dopamine agonist drugs have direct stimulatory effect on the pancreatic β cells with consequent improvement of insulin release in response to the blood glucose. However, glucose loaded inhibits insulin secretion in Parkinson disease patients treated with L‐dopa. Chronic hyperprolactinaemia affects the metabolic rate of adipose tissue, liver, skeletal muscles, and pancreas causing alteration of insulin signalling and development of IR and hyperglycaemia (Figure 6).^89^

Moreover, impairment of lipid profile is linked with PRL excess in patients with hyperprolactinaemia.^90^ Body fat percentage in conjugation with dyslipidemia is augmented in patients with hyperprolactinaemia compared with healthy controls.^90^ In particular, triglyceride, cholesterol, and LDL serum levels are increased while HDL serum level is reduced in patients with hyperprolactinaemia compared to healthy controls.^91^ Besides, apolipoprotein A and B are reduced in women with hyperprolactinaemia. It has been shown that PRL has direct effect on the differentiation of adipose tissue due to higher expression of PRLRs in these sites.^92^ Moreover, dopaminergic tone which affects the metabolic rate of adipose tissue due to higher expression of dopamine receptors in the adipose tissues, inhibit PRL signalling in adipose tissues.^93^ Therefore, dopaminergic agonist agents may improve adipose tissue function by inhibiting local adipose tissue PRL and improve lipid regardless of body weight and visceral mass signifying direct and indirect effects of dopaminergic agonists on lipid profile. In addition, circulating PRL is involved in regulating lipid storage in the liver, and serum PRL was reported to be reduced in patients with non‐alcoholic fatty liver disease and hepatic steatosis as compared to healthy controls.^94^ Therefore, hyperprolactinaemia‐induced dyslipidemia is developed due to multiple mechanistic pathways including reduction of lipoprotein lipase activity, hypogonadism, weight gain, and GH deficiency.

Thus, PRL excess as in hyperprolactinaemia may induce the development and progression of MetS by different mechanistic pathways.

## 7 DISCUSSION

Findings of the present review highlighted that both low and high PRL levels are associated with metabolic disorders and development of MetS as consistence with other studies.^28^, ^95^ Although PRL levels are mainly depending on age, sex and physiological status, it has been shown that maintenance of metabolically and biologically active beneficial PRL level is essential to prevent the development and progression of MetS.^60^ A metabolic beneficial PRL level within 25–100 μg/L is called HomeoFIT‐PRL.^60^ However, an updated review suggests that HomeoFIT PRL within 7–100 μg/L could prevent metabolic disease development, whereas too‐low and too‐high PRL levels are associated with an increased prevalence of metabolic diseases.^96^ Therefore, a new classification of PRL level is mandatory to fit the metabolic effect. For example, PRL level >200 μg/L is correlated with prolactinoma whereas PRL level <25 μg/L is regarded as normal.^95^ However, transient and reactive hyperprolactinaemia as in stress, exercise, sexual function and hypoglycaemia are regarded as a physiological response for the adaptation.^97^ Of note, upper‐limit PRL level within the physiological range seems to be a compensatory mechanism to mitigates inflammatory and oxidative stress as in obesity.^82^ However, sever hyperprolactinaemia is associated with IR, hyperglycaemia and hyperinflammation in patients with prolactinoma compared to healthy subjects.^82^ Hyperprolactinaemia in mice with high‐fat induced obesity, aggravates the metabolic disturbances by inhibiting thermogenesis in brown adipose tissue.^98^ In addition, hyperprolactinaemia due to primary hypothyroidism is also associated with metabolic disturbance and may increase MetS risk.^99^ Therefore, reduction of PRL levels by dopaminergic agonists is essential to prevent metabolic disorders in patients with hyperprolactinaemia. However, intensive treatments with dopaminergic agonists may reduce PRL levels beyond the physiological levels causing metabolic disturbances.^28^, ^60^ Thus, appropriate treatment of hyperprolactinaemia to restore normal PRL levels in patients with prolactinoma is mandatory therapeutic strategy. However, macroprolactinoma with symptomatic mass effects should be treated according to the size of prolactinoma regardless of PRL serum levels.^100^ Furthermore, postmenopausal hyperprolactinaemia is linked with oestrogen deficiency and may cause severe cardiometabolic complications. Thus, decision to start or stop treatment with dopaminergic agonists in postmenopausal hyperprolactinaemia is mainly depending on the metabolic profile.^101^

The beneficial effects of HomeoFIT‐PRL against MetS are related to its antioxidant and anti‐inflammatory effects, attenuation of mitochondrial dysfunction that reduces inflammatory and oxidative disorders in MetS.^102^ The mechanisms that mediate PRL effects on β‐cells involve increased osteoprotegerin synthesis, leading to the inhibition of receptor activator of NF‐kB ligand pathway, an inhibitor of β‐cell proliferation, increased survivin levels, promotes the expression of the transcription factors Foxm1 and MafB, increased cyclin activity, and higher islet serotonin production through Tph1 synthesis, all promoting β‐cell proliferation. Also, PRL leads to the inhibition of extrinsic and intrinsic apoptosis pathways and improved glucose sensitivity through increased glucokinase and the expression of GLT2.^96^ Moreover, activation of STAT5 downstream of the PRLR facilitates the insulin sensitising effects of PRL. PRLR interacts with insulin substrate 1 (IRS1) and promotes the phosphorylation of AKT, two key members of the insulin signalling pathway.^103^

It has been shown that resveratrol reduces biomarkers of inflammatory and oxidative disorders in patients with MetS.^104^ Notoriously, resveratrol inhibits proliferation of prolactinoma and restores normal PRL levels.^104^ Likewise, cAMP activators like β‐adrenoceptor agonists increase expression and release of PRL from preadipocytes.^105^ Notably, β‐adrenoceptor agonists improve metabolic profile in MetS.^106^ Therefore, increasing of PRL level to its upper limit may prevent metabolic derangement and development of MetS.

Taken together, PRL deficiency and excess is associated with development of MetS, though the underlying mechanisms for this association is not fully elucidated.

## 8 CONCLUSIONS AND PERSPECTIVES

The development of MetS is affected by PRL hormone which affect the development of IR and central obesity. Pituitary PRL controls mammary gland, however extra‐pituitary PRL is highly intricate in the regulation of adipose tissue function. Normal physiological level of PRL is essential for insulin sensitivity and regulation of adipose tissue function and energy metabolism. Normal physiological PRL levels seem beneficial against IR, obesity and development of MetS. However, PRL excess as in hyperprolactinaemia may induce the development and progression of MetS by different mechanistic pathways. Thus, HomeoFIT‐PRL is considered as an appropriate PRL level that prevents the development of MetS. Findings of the present review highlighted that both low and high PRL levels are associated with metabolic disorders and development of MetS as consistence with other studies.^28^, ^95^ The beneficial effects of HomeoFIT‐PRL against MetS are related to its antioxidant and anti‐inflammatory effects, attenuation of mitochondrial dysfunction that reduces inflammatory and oxidative disorders in MetS.^102^ The mechanisms that mediate PRL effects on β‐cells involve increased osteoprotegerin synthesis, leading to the inhibition of receptor activator of NF‐kB ligand pathway, an inhibitor of β‐cell proliferation.^95^ Also, PRL leads to the inhibition of extrinsic and intrinsic apoptosis pathways and improved glucose sensitivity through increased glucokinase and the expression of GLUT2.^96^ Moreover, activation of STAT5 downstream of the PRLR facilitates the insulin sensitising effects of PRL. PRLR interacts with insulin substrate 1 (IRS1) and promotes the phosphorylation of AκT, two key members of the insulin signalling pathway.^103^

Taken together, appropriate circulating PRL is essential for regulation of adipose tissue metabolism and insulin action. Searching for specific PRLR agonists may be effective in treating cardiometabolic disorders associated with MetS in patients with low circulating PRL. However, dopamine agonists could be effective in treating hyperprolactinaemia‐induced MetS. Preclinical and clinical studies are recommended in this regard.

## AUTHOR CONTRIBUTIONS

**Ayah Talal Zaidalkilani:** Writing – review and editing (equal). **Hayder M. Al‐Kuraishy:** Conceptualization (equal); data curation (equal); formal analysis (equal); funding acquisition (equal); investigation (equal); methodology (equal); project administration (equal); resources (equal); software (equal); supervision (equal); validation (equal); visualization (equal); writing – original draft (equal); writing – review and editing (equal). **Ali I. Al‐Gareeb:** Conceptualization (equal); data curation (equal); formal analysis (equal); funding acquisition (equal); investigation (equal); methodology (equal); project administration (equal); resources (equal); software (equal); supervision (equal); validation (equal); visualization (equal); writing – original draft (equal); writing – review and editing (equal). **Athanasios Alexiou:** Conceptualization (equal); data curation (equal); formal analysis (equal); funding acquisition (equal); investigation (equal); methodology (equal); project administration (equal); resources (equal); software (equal); supervision (equal); validation (equal); visualization (equal); writing – original draft (equal); writing – review and editing (equal). **Marios Papadakis:** Conceptualization (equal); data curation (equal); formal analysis (equal); funding acquisition (equal); investigation (equal); methodology (equal); project administration (equal); resources (equal); software (equal); supervision (equal); validation (equal); visualization (equal); writing – original draft (equal); writing – review and editing (equal). **Ammar AL‐Farga:** Writing – review and editing (equal). **Othman A. Alghamdi:** Writing – review and editing (equal). **Mostafa M. Bahaa:** Conceptualization (equal); data curation (equal); formal analysis (equal); funding acquisition (equal); investigation (equal); methodology (equal); resources (equal); software (equal); supervision (equal); validation (equal); visualization (equal); writing – original draft (equal); writing – review and editing (equal). **Gaber El‐Saber Batiha:** Conceptualization (equal); data curation (equal); formal analysis (equal); funding acquisition (equal); investigation (equal); methodology (equal); project administration (equal); resources (equal); software (equal); supervision (equal); validation (equal); visualization (equal); writing – original draft (equal); writing – review and editing (equal). **Mohammed S. Alshammari:** Writing – review and editing (equal). **Mohammed Alrouji:** Writing – review and editing (equal).

## FUNDING INFORMATION

Open access funding enabled and organized by Projekt DEAL. This work was supported by University of WITTEN/HERDECKE, Germany.

## CONFLICT OF INTEREST STATEMENT

The authors have no conflict of interest to declare.

## Data Availability

All data are available in the manuscript.
